# Death of Dr. Beaumont

**Published:** 1853-08

**Authors:** 


*Death of Dr. Beaumont.*—Dr. Beaumont, the author of the justly celebrated researches on the subject of Digestion in a case of fistulous opening in the stomach, has recently died at St. Louis, aged about 68 years.

